# Selective Modulation of TNF–TNFRs Signaling: Insights for Multiple Sclerosis Treatment

**DOI:** 10.3389/fimmu.2018.00925

**Published:** 2018-04-30

**Authors:** Valentina Pegoretti, Wia Baron, Jon D. Laman, Ulrich L. M. Eisel

**Affiliations:** ^1^Department of Molecular Neurobiology (GELIFES), University of Groningen, Groningen, Netherlands; ^2^Department of Cell Biology, University Medical Center Groningen (UMCG), University of Groningen, Groningen, Netherlands; ^3^Department of Neuroscience, University Medical Center Groningen (UMCG), University of Groningen, Groningen, Netherlands

**Keywords:** tumor necrosis factor alpha, TNFR2, TNFR1, immune tolerance, multiple sclerosis, neurodegeneration

## Abstract

Autoimmunity develops when self-tolerance mechanisms are failing to protect healthy tissue. A sustained reaction to self is generated, which includes the generation of effector cells and molecules that destroy tissues. A way to restore this intrinsic tolerance is through immune modulation that aims at refurbishing this immunologically naïve or unresponsive state, thereby decreasing the aberrant immune reaction taking place. One major cytokine has been shown to play a pivotal role in several autoimmune diseases such as rheumatoid arthritis (RA) and multiple sclerosis (MS): tumor necrosis factor alpha (TNFα) modulates the induction and maintenance of an inflammatory process and it comes in two variants, soluble TNF (solTNF) and transmembrane bound TNF (tmTNF). tmTNF signals *via* TNFR1 and TNFR2, whereas solTNF signals mainly *via* TNFR1. TNFR1 is widely expressed and promotes mainly inflammation and apoptosis. Conversely, TNFR2 is restricted mainly to immune and endothelial cells and it is known to activate the pro-survival PI3K-Akt/PKB signaling pathway and to sustain regulatory T cells function. Anti-TNFα therapies are successfully used to treat diseases such as RA, colitis, and psoriasis. However, clinical studies with a non-selective inhibitor of TNFα in MS patients had to be halted due to exacerbation of clinical symptoms. One possible explanation for this failure is the non-selectivity of the treatment, which avoids TNFR2 stimulation and its immune and tissue protective properties. Thus, a receptor-selective modulation of TNFα signal pathways provides a novel therapeutic concept that might lead to new insights in MS pathology with major implications for its effective treatment.

## Introduction

Multiple sclerosis (MS) affects approximately 2.5 million people worldwide. It is considered as an autoimmune disease characterized by white and gray matter lesions in the central nervous system (CNS) caused by autoreactive T cells that escaped from central and peripheral tolerance patrolling mechanisms. These cells travel along with activated B cells and monocytes to the CNS where they infiltrate, starting a synergistic attack against myelin (1). As demyelination is a key feature of MS pathology, several myelin proteins have been investigated as targets of these autoreactive lymphocytes. It has been shown that myelin basic protein and myelin oligodendrocyte glycoprotein are recognized by mature autoreactive T-helper cells in MS patients but also in healthy individuals (2). The identification of a major T cell autoantigen in MS is still a matter of controversy. It may be due to technical limitations in autoantibodies’ detection or epitope spreading (3). Anyway, the search of pathological anti-myelin immune responses is still open-ended.

Currently, the etiology of MS has been investigated from another angle that favors the idea that initial pathology occurs within the CNS, similarly to other neurodegenerative disorders such as Alzheimer’s and Parkinson’s diseases (4). This theory argues that degeneration of oligodendrocytes and/or myelin initiates pathology by releasing autoantigen, which in turn are responsible for the autoimmune, inflammatory response in the organism. Of importance, mitochondrial dysfunction (5), ROS production (6), misfolding of proteins (7), and release of proapoptotic signals (8) are just few of the consequences (9). Myelin-loaded microglia/macrophages are also largely involved in this pathological process (10). They are constantly producing ROS through oxidative burst giving rise to mitochondrial dysfunction and proapoptotic signals release causing oligodendrocytes death and demyelination.

Being it autoimmune or neurodegenerative, the study of the nature of this disease is yet mostly descriptive than causative. With this limited understanding, the animal models currently available seem to mimic only few and separated features of the disease, which further restrict our view of the underlying mechanisms causing MS. Even though it seems very difficult to achieve a solid and unifying explanation, great efforts have been made to develop treatments to reduce the symptomatic incidences in MS patients. The available therapeutic strategies are primarily focused on suppressing or modulating certain immune functions thereby leading to a partial and temporary recovery sometimes with major side effects (11). There is still a strong urge for an effective treatment that slows down MS disease progression or to prevent its development.

This review will focus on the potential value for MS treatment of tumor necrosis factor alpha (TNFα), a major cytokine involved in several biological functions. Furthermore, the different and dual functions of TNFα are specified by the two receptors (TNFR1 and TNFR2) that it activates. Current research highlights a great potential of selectively targeting TNF–TNFRs signaling with promising immune protective, tissue regenerative, and neuroprotective therapeutic properties.

## Anti-TNF Therapies in Autoimmunity

Self-recognition is an essential biological process that gives rise to immune tolerance: a state of indifference or non-reactivity toward a substance that would normally be expected to excite an immunological response (12). Yet, when the immune system erroneously identifies a self-antigen as a danger, it initiates an inflammatory response against it. The latter mechanism is defined as *autoimmunity*, which encompasses tissue damage, caused by T-cell or antibody reactivity to self. Many inflammatory diseases are autoimmune diseases, including rheumatoid arthritis (RA), MS, Graves’ disease, type 1 diabetes mellitus, Crohns’ disease, and others. Nowadays, they affect 12.5% of the world’s population (13) and they can be distinguished based on their primary target organ (joints, skin—psoriasis; CNS—MS; intestine—inflammatory bowel disease (IBD); pancreas—type 1 diabetes mellitus). For many years, the standard treatment relied on diminishing autoimmune pathology with general immunosuppressive agents, anti-proliferative drugs, and corticosteroids. Halting the immune system has always major and diffuse side effects that increase the toxicity of the intervention thereby decreasing its therapeutic value. Immunosuppressant drugs are widely used by clinicians to reduce inflammatory attacks on tissues but, due to their low efficacy, disease-modifying drugs with greater specificity and lower toxicity were implemented. Monoclonal antibodies and engineered biological products have become now standard interventions for several autoimmune diseases, including MS. A long standing class of biologics used for many autoimmune diseases is TNF blockers which includes infliximab, etanercept, adalimumab, PEGylated certolizumab, and golimumab. These are the FDA-approved anti-TNF biologics for the treatment of Crohn’s disease, ulcerative colitis, RA, ankylosing spondylitis (AS), psoriatic arthritis, and plaque psoriasis (14). Even though it has been extensively studied, the potential therapeutic value of blocking TNF is limited by its partial efficacy in different diseases. Anti-TNF treatment is discontinued in 1/3 of RA patients within the first year of treatment (15). Around 10–30% of IBD patients do not respond to initial treatment while 23–46% lose response over time (16). Similarly, 27% of patients with psoriasis discontinue anti-TNF treatment after a year or lose its efficacy over time (17). So far, there is little evidence explaining the reasons and risk factors for primary or secondary non-response. Therefore, other strategies are implemented by clinicians to maintain efficacy with acceptable tolerability such as using a different TNF blocker, switching class of biologic, dose adjustments, and change in route of administration, when possible. Moreover, failure of anti-TNF therapies can also be due to development of adverse effects such as infections, malignancies, acute infusion and injection reactions, autoimmunity, and cardiovascular effects (18, 19).

In 1999, a clinical trial testing the efficacy of a TNF inhibitor, Lenercept, for MS treatment had to be halted due to exacerbations of symptoms when compared to placebo-treated MS patients (20). Likewise, there are several clinical reports of RA and AS patients treated with TNF blockers that developed CNS demyelination after treatment (21, 22).

**Figure 1:**
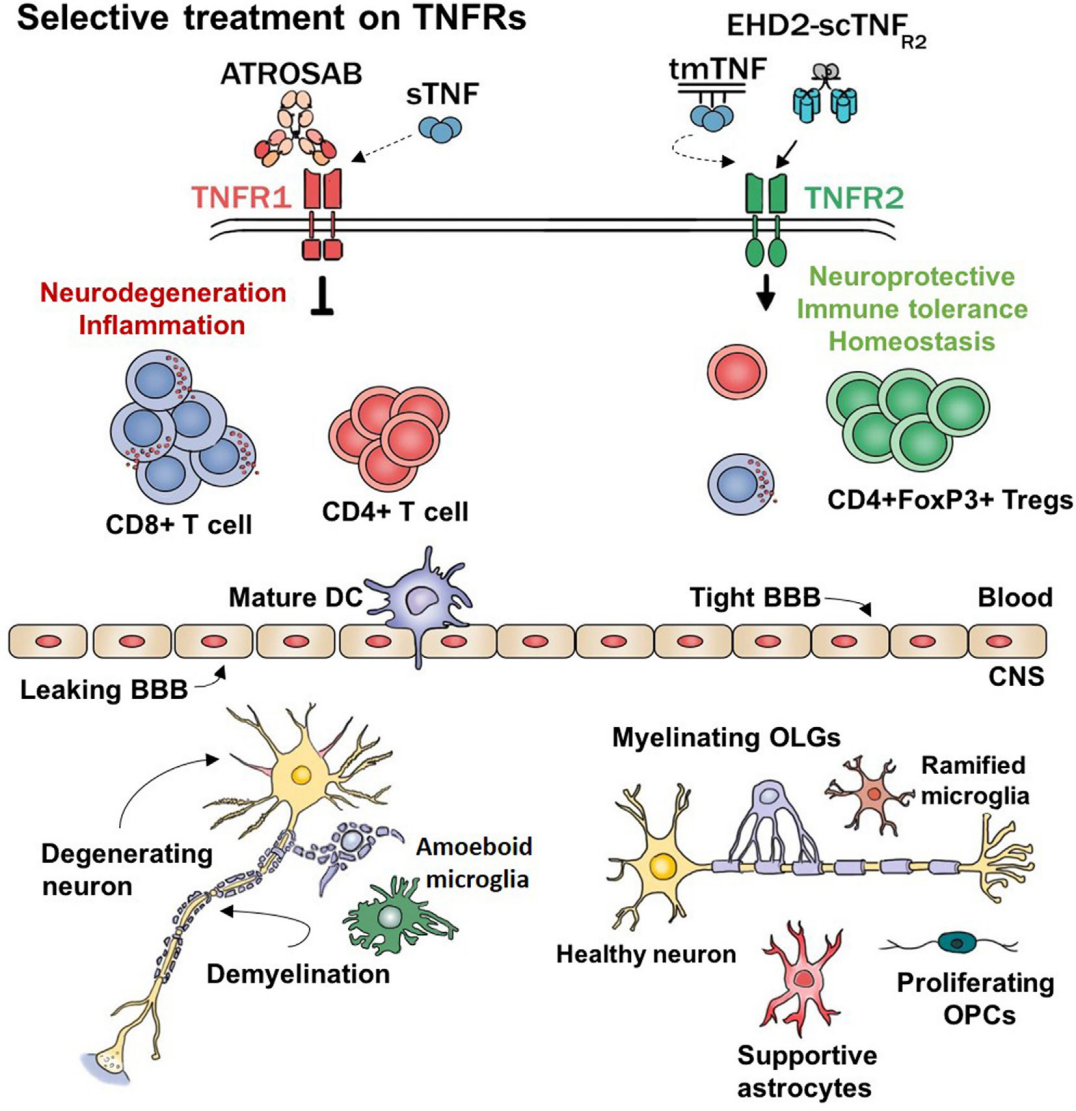
Hypothetical working model. TNFα and its receptors regulate major functions of several cell types. This model represents the expected effects of selectively modulating TNF–TNFRs signaling. sTNF, soluble TNF; tmTNF, transmembrane TNF; ATROSAB, TNFR1 antagonistic antibody; EHD2-scTNFR2, TNFR2 agonist; CD8^+^ T cell, cytotoxic T cells; CD4^+^ T cells, helper T cells; DC, dendritic cell; BBB, blood–brain barrier; Tregs, regulatory T cells; CNS, central nervous system; OPCs, oligodendrocyte’s precursor cells; OLGs, oligodendrocytes; TNFα, tumor necrosis factor alpha.

Although partially effective in other autoimmune diseases, anti-TNF therapies in MS patients seem to worsen pathology and clinical symptoms. A possible explanation for this failure is the inability of the drug to grant access to the CNS (23). In other tissues is rather easy to penetrate and exert a local effect, the brain is a privileged site that instead restricts entry to macromolecules such as biologics. Furthermore, non-selective TNF inhibitors dampen down the active inflammatory response ongoing in certain diseases such as RA and IBD. While for these diseases the anti-inflammatory effects could be enough for (at least partial) recovery, MS treatment requires a more profound reestablishment of homeostasis that includes tissue protective and regenerative properties. Interestingly, the last two decades of research on TNFα signaling showed that the soluble form of TNF (solTNF) triggers apoptotic and proinflammatory signals to the cell *via* TNFR1 while the transmembrane form (tmTNF) is able to promote cell survival through TNFR2 activation (see Figure 1). The following chapter recapitulates the current studies trying to specify and optimize selectively targeting TNF–TNFRs within an MS therapeutic frame.

## TNF–TNFR Signaling: Therapeutic Implications for MS

Tumor necrosis factor alpha is a pleiotropic cytokine regulating many physiological and pathological functions such as cell survival (24), apoptosis (25), inflammation (26–28), autoimmunity (29), demyelination (30), and cancer (31). TNFα is synthesized as a transmembrane protein of 26 kDa and forms a stable homo-trimeric molecule (tmTNF). Proteolytic cleavage of the protein *via* TNFα converting enzyme (TACE/ADAM17) produces a 17-kDa monomeric protein, a soluble homo-trimeric molecule of 51 kDa (solTNF). TNFα signaling is then generated through the interaction with two distinct transmembrane receptors, the 55-kDa TNF receptor type I (TNFR1) and the 75-kDa TNF receptor type II (TNFR2). The two TNFα variants display different affinities for the two receptors. TNFR1 is activated by both soluble and transmembrane forms with higher affinity for solTNF while activation of TNFR2 is solely due to tmTNF. Furthermore, the two receptors differ in the intracellular pathways that they trigger leading to various cellular responses (32–34). TNFR1 has been described as stimulator of effector caspase-mediated apoptosis (35, 36), while TNFR2 promotes cell survival through PI3K-Akt/PKB signaling pathway (37, 38). However, TNFR1 activation may also prevent TNF-induced apoptosis by activating the classical NF-κB pathway (39) and receptor-interacting protein 1 (RIP1) ubiquitination (40). Upon TNFR1 stimulation, the intracellular death domain (DD) recruits RIP1 and TNFR-associated death domain (TRADD). TRADD engages TNFR-associated factor 2 (TRAF2), inhibitor of apoptosis protein 1 (cIAP1) and inhibitor of apoptosis protein 2 (cIAP2) thereby leading to the formation of complex I (41). RIP1 ubiquitination and complex I activation later stimulates catalytic IκB kinase (IKK) complex, which favors the activation of NF-κB pathway (42). If this signaling fails, complex II will trigger caspase 8-mediated apoptosis upon TNFR1 ligand binding (43). Importantly, the initiation of this apoptotic process heavily relies on the levels of the inhibitory protein (cFLIP). The more NF-κB is activated by complex I, the more cFLIP will be available to inhibit caspase-mediated apoptosis (44).

In addition, it was shown that TNFRs cross talk intracellularly giving rise to TNFR1-induced cell survival and TNFR2-induced apoptosis (33). In contrast with TNFR1, TNFR2 does not contain a DD but it is still capable of inducing apoptosis upon its activation (45). A common intracellular molecule family in the TNFα signaling cascade is TRAFs, which are recruited by both TNFRs complexes. In CD30 and CD40 cells, TNFR2 stimulation might lead to TRAF2 degradation, which results in caspase 8 activation and eventually apoptosis (46). TRAF2 is also an important regulator of cell survival through TNFR1-mediated activation of C-Jun and NF-κB. *In vitro* studies showed that NF-κB activation leads to production of TRAF1, which blocks TNFR2-mediated degradation of TRAF2 (47).

Another distinctive feature of these two receptors is their differential expression in different tissues. While TNFR1 is ubiquitously expressed, TNFR2 can be found mainly on endothelial cells, various immune cells, and certain CNS cells (48).

All these peculiar features enable such a complex cytokine to have major, sometimes conflicting, effects depending on its form, the receptor that it triggers and the cell type on which it may act (see Figure 1). Because of this pleiotropic effect, the function of TNFα will depend on the ratio of co-expression of its receptors which will shift the balance between cellular survival and apoptosis.

The following sections will highlight the beneficial properties of targeting selectively TNFRs’ signaling pathway found in *in vitro* and *in vivo* models of MS (see Table 1). This may help to further elucidate the therapeutic value of TNFα in the treatment of MS and other autoimmune diseases.

**Table 1:** Animal models to investigate pharmacological interventions for multiple sclerosis (MS).

Models	Species	Induction	Mechanism of action	Effect on physiology	Relevance	Relevance for TNFRs selective approach
EAE model (49)	Rodents, rabbit, primate	Immunization	Autoimmune reaction vs. myelin protein	T cell dysfunction	Autoimmunity	To study the anti-inflammatory effects against T cell autoreactivity and immune protection through Tregs
Cuprizone model (50)	Rodents	Toxic	Unknown. Iron chelator causing mt dysfunction in OLGs	Degeneration of OLGs, mainly in CC	Myelin degeneration and regeneration	To study the (re)generative properties on myelin components, OPCs and OLGs
NBM lesion model (51)	Rodents	IC injections of NMDA	Neuronal excitotoxicity	Neuronal degeneration in NBM	Neurodegeneration and/or protection	To study the neuroprotective properties on axonal loss, apoptosis, mt dysfunction and signal transmission

*Selectively targeting TNFR1 and/or TNFR2 has promising therapeutic potential. The animal models available nowadays have several limitations (52, 53) that require the study of different pathological hallmarks of disease in different models. Here, we show three widely used animal models that give an almost exhaustive overview of MS pathology when taken altogether*.

*EAE, experimental autoimmune encephalomyelitis; NBM, nucleus basalis of Meynert; IC, intracerebral; NMDA, N-methyl-d-aspartate acid; MT, mitochondrial; OLG, oligodendrocyte; CC, corpus callosum; OPC, oligodendrocytes precursors cell; Tregs, regulatory T cell*.

## Selective Targeting of TNFRs: Immune Protection Properties

Growing evidence suggest that proinflammatory factors are intertwined with a complex resolution program of inflammation after few hours an inflammatory response begins (54–56). In a coculture experiment with murine CD4^+^CD25^+^FoxP3^+^ regulatory T cells (Tregs) and CD4^+^CD25^−^ effector T cells (Teffs), short-term exposure to TNFα promotes Teffs expansion while a more prolonged treatment favors proliferation and activation of Tregs (57). Moreover, TNFR2^−/−^ mice show normal pool of Tregs but, when stimulated with septic challenge, they fail to expand. This suggests that TNFα might have a proinflammatory action in the early phases of inflammation through Teffs to leave then Tregs to re-establish homeostatic balance through TNFR2 signaling. Similar evidence comes from leukocytes isolated from RA patients on anti-TNF medications (58). Adalimumab, an anti-TNF antibody, but not the solTNFR2 Etanercept, promotes the interaction between monocytes and Tregs leading to expansion of FoxP3^+^ Tregs and suppression of Th17 cells through IL-2/STAT5 pathway. This effect is caused by adalimumab binding to tmTNF on monocytes, which is able to enhance both expression of tmTNF and its binding to TNFR2 on Tregs. Additionally, increased IL-17 production in TNFR2-deficient T cells is prevented by exogenous IL-2 showing that tmTNF–TNFR2 signaling suppresses Th17 differentiation by promoting IL-2 expression (59). Furthermore, an *in vivo* EAE study with TNFRs^−/−^ mice showed a reduction of clinical symptoms, demyelination score, CD3^+^ T cell infiltrates, and activated microglia/macrophages in TNFR1^−/−^ mice. On the contrary, lacking TNFR2 seems to worsen EAE disease course. In the same study, EAE was induced in normal C57BL/6 to investigate the effect of antibody-mediated TNFR1 inhibition. These results show attenuated EAE severity and delayed the onset of disease in the treated group mainly through decreased demyelination score and neuronal loss while there is only a mild reduction in immune infiltrates into the CNS (60). If silencing TNFR1 is not enough, activation of TNFR2 in mouse microglia culture promotes expression of anti-inflammatory and neuroprotective genes as granulocyte colony-stimulating factor, adrenomedullin, IL-10, and IFN-γ (61). Specifically, conditional knockout of microglial TNFR2 reveals earlier EAE onset by means of increased number of infiltrates, T cell activation, and demyelination scores. On the other hand, ablation of microglial/macrophage TNFR2 leads to EAE suppression (62). This experiment further expands our knowledge of TNFα functions on its receptors: TNFR2 has dual roles depending on its location in central or peripheral myeloid cells as much as solTNF and tmTNF have detrimental or protective properties, respectively.

## Selective Targeting of TNFRs: Tissue Regeneration Properties

A crucial pathological hallmark of MS is white matter lesions caused by axonal demyelination. An effective pharmacological intervention for this disease requires tissue regenerative properties to counteract tissue damage at the lesion site. *In vitro* studies reveal that TNFR2 activation protects oligodendrocyte’s progenitor cells (OPCs) from oxidative stress (63). OPCs are increasingly being studied in MS research as they are shown to be essential to the remyelination process (64). The beneficial effect of TNFR2 seems to continue in later stages of development of these critical cells. In a primary coculture setup, maturation of oligodendrocytes into myelinating cells appears to be boosted through astrocyte-specific TNFR2 stimulation (65). In 2001, an important *in vivo* study investigated the different role of TNFRs in the cuprizone model for demyelination (see Table 1) in mice lacking either TNFα or one its receptors (66). In this study, the absence of TNFα delays the remyelination process due to reduction of proliferating OPCs and mature oligodendrocytes when compared to wild type mice. Interestingly, similar effects are found in mice lacking TNFR2, but not TNFR1, underling a substantial role of TNFR2 in promoting oligodendrocytes proliferation and regeneration. In this vein, inhibition of solTNF shows that tmTNF increases axon preservation and improves myelin compaction in an EAE mouse model for MS (67). In the same study, myelin-specific genes and increased number of OPCs are found upon tmTNF treatment. A recent EAE study with conditional knockout mice highlights that TNFR2 specifically on oligodendrocytes drives their differentiation and remyelination (68). Furthermore, treatment with XPro1595, a selective solTNF inhibitor, in a cuprizone mouse model (see Table 1) shows faster remyelination due to improved myelin phagocytosis by microglia (69).

## Selective Targeting of TNFRs: Neuroprotection Properties

In the progressive stages of MS, axonal loss and neurodegeneration seem to take over, at least partially, inflammation as main pathological hallmarks (70). Several *in vitro* studies underline the potential neuroprotective effect of selective targeting of TNFRs. In a human dopaminergic neuronal cell line (LUHMES), TNFR2 stimulation of the PI3K-PKB/Akt pro-survival pathway rescues neurons from oxidative stress-induced cell death (71). Furthermore, similar results were found in an *in vitro* model of glutamate-induced excitotoxicity in primary cortical neurons. TNFR2, and not TNFR1, induces persistent PI3K PKB/Akt-mediated NF-κB activation leading to neuroprotection, which is enhanced by *N*-methyl-d-aspartate receptor co-stimulation (37). Using the same *in vitro* model, another study shows that activation of TNFR2 signaling pathway mediated lovastatin-induced neuroprotection against glutamate excitotoxicity (72). Statins are widely prescribed in clinical practice for lowering cholesterol levels. Nonetheless, a specific statin, called Simvastatin, has been shown to be effective in decreasing whole-brain atrophy in patients with secondary progressive MS in a phase II trial (73). The neuroprotective effect of TNFR2 was also found in an *in vivo* model using TNFR1^−/−^ and TNFR2^−/−^ mice. After retinal ischemia, TNFR1 deficiency leads to strong decrease in neuronal death while absence of TNFR2 leads to enhanced neurodegeneration (36). Another *in vivo* model using genetic ablation of solTNF shows neuroprotection against focal cerebral ischemia (74). Interestingly, a recent study reveals that the neuroprotective and anti-inflammatory effects found by antagonizing TNFR1 in the nucleus basalis lesion (NBM) model (see Table 1) is enhanced through TNFR2 signaling (75).

## Future Perspectives

Due to contradicting results concerning TNFα and TNFR signaling in neurodegenerative diseases in the late 90s, major advances have been made in recent years in understanding the biology of TNF–TNFRs signaling in health and disease. This review highlighted the potential therapeutic value of this target, specifically within MS pathology. Of importance, the available MS treatments are focused on limiting the burden and occurrence of autoreactive peripheral immune cells. We can see them as drugs boosting the immune system’s resistance toward an insult against self-tissue. Obviously, this leads to a temporary effect of the treatment, which is followed by a partial decrease in symptoms, mainly in patients with relapsing-remitting MS. Moreover, most of these drugs are not able to slow down disease progression. Recently, several immunologists and evolutionary ecologists introduced the concept of *disease tolerance* as a defense mechanism against infectious agents (76–78). In flies (79), rodents (80), and humans (81) studies, modulating disease tolerance resulted in protection against several types of infection and restored homeostasis (82). As in different patrolling mechanisms, attack is not always the best defense mechanism: damage control is as important as pathogen control. Within autoimmunity, dysregulated disease tolerance can be seen as a failure of the immune system to control tissue damage caused by autoreactive immune cells. Interestingly, selective modulation of TNFRs triggers a variety of protective and pro-survival properties, which in turn are positively affecting the pathological milieu derived from autoreactive lymphocytes. Breaking the vicious circle of chronic inflammation and protect tissue against further damage are essential features for a therapeutic agent that aims at restoring proper immune functions and general homeostasis.

Nevertheless, some challenges need to be addressed to further elucidate the potential of this treatment target. As briefly mentioned above, activating TNFR2 in peripheral or central myeloid cells resulted in opposing therapeutic effects (62) underling the need for a pharmacological approach that minimizes peripheral immune activation. However, blood–brain barrier (BBB) permeability of these compounds might be an obstacle to overcome in order to reach all beneficial effects of this target. In the past decade, great progress has been made in developing nanoparticles (83, 84) and cell-specific drug carriers (85) through the BBB, giving a promising perspective for CNS diseases (86).

To conclude, selective modulation of TNFRs through TNFR2 activation and/or TNFR1 silencing has great therapeutic potential in terms of immune, tissue, and neuroprotective properties, especially for MS treatment.

## Author Contributions

The authors contributed equally to this work.

## Conflict of Interest Statement

The authors declare that the research was conducted in the absence of any commercial or financial relationships that could be construed as a potential conflict of interest.
